# Tumor-Associated Macrophages Induce Migration of Renal Cell Carcinoma Cells via Activation of the CCL20-CCR6 Axis

**DOI:** 10.3390/cancers12010089

**Published:** 2019-12-30

**Authors:** Suguru Kadomoto, Kouji Izumi, Kaoru Hiratsuka, Taito Nakano, Renato Naito, Tomoyuki Makino, Hiroaki Iwamoto, Hiroshi Yaegashi, Kazuyoshi Shigehara, Yoshifumi Kadono, Hiroki Nakata, Yohei Saito, Kyoko Nakagawa-Goto, Atsushi Mizokami

**Affiliations:** 1 Department of Integrative Cancer Therapy and Urology, Kanazawa University Graduate School of Medical Science, Kanazawa 920-8641, Japan; 32f3k8@bma.biglobe.ne.jp (S.K.); hirakaoru@med.kanazawa-u.ac.jp (K.H.); urotaito@yahoo.co.jp (T.N.); thealfuu@yahoo.co.jp (R.N.); mackeeen511@gmail.com (T.M.); hiroaki017@yahoo.co.jp (H.I.); hyae2002jp@yahoo.co.jp (H.Y.); kshigehara0415@yahoo.co.jp (K.S.); yskadono@yahoo.co.jp (Y.K.); mizokami@staff.kanazawa-u.ac.jp (A.M.); 2 Department of Histology and Cell Biology, Kanazawa University Graduate School of Medical Science, Kanazawa 920-8641, Japan; hnakata@staff.kanazawa-u.ac.jp; 3 School of Pharmaceutical Sciences, College of Medical, Pharmaceutical and Health Science, Kanazawa University, Kanazawa 920-1192, Japan; saito-y@staff.kanazawa-u.ac.jp (Y.S.); kngoto@p.kanazawa-u.ac.jp (K.N.-G.)

**Keywords:** CCL20, CCR6, migration, renal cell carcinoma, tumor-associated macrophages

## Abstract

This study investigated tumor-associated macrophages activity in the microenvironment of renal cell carcinoma. Via a co-culture with macrophage-like cells differentiated from human monocyte cell line THP-1 and U937 cells, the migration ability of ACHN and Caki-1 cells, which are human renal cell carcinoma cell line cells, was significantly increased, as was the epithelial–mesenchymal transition change. A chemokine array identified the CCL20-CCR6 axis as a concentration-dependent signal in ACHN and Caki-1 cell migration. Akt in the ACHN and Caki-1 cells was activated by macrophage-like cells, and the CCL20 neutralizing antibody suppressed migration ability, epithelial–mesenchymal transition, and Akt phosphorylation in the ACHN and Caki-1 cells. Akt inhibitor AZD5363 also decreased the epithelial–mesenchymal transition change and migration ability in the ACHN and Caki-1 cells. In 42 renal cell carcinoma tissues, patients with CCR6 and macrophage infiltration indicated poor prognoses. In the tumor microenvironment of renal cell carcinoma, cancer cells are activated by CCL20 secreted by tumor-associated macrophages through Akt activation, followed by epithelial–mesenchymal transition and an acquired migration ability. Thus, inhibition of the CCL20-CCR6 axis may be a potential therapeutic strategy for renal cell carcinoma.

## 1. Introduction

The most dominant histological phenotype of kidney cancer is renal cell carcinoma (RCC), which accounts for 3–4% of all cases of adult cancer in the United States [1]. A total of 20–40% of RCC patients have recurrent metastatic disease after a primary nephrectomy; therefore, approximately 50% of all patients who are diagnosed with RCC should receive systemic therapy during the course of their disease [2]. The prognosis of advanced and metastatic RCC is not satisfactory, even with the recent evolution of treatment methods [3]. RCC is classified into many subtypes, including clear cell RCC, which accounts for 70% of all RCC [4]. As clear cell RCC shows immunogenicity, immunotherapy, such as interferon-α and interleukin-2, played a central role in RCC treatment until the 1990s [5]. Many studies have shown the involvement of immune cells that induce immune tolerance in the progression of RCC, and tumor-associated macrophages (TAMs) have been found to be an important element of the tumor microenvironment of RCC [6]. Although normal M1-type macrophages may work to inhibit cancer cell activity, TAMs, which are representative of M2-type macrophages, are involved in the promotion of cancer cell growth, invasion, angiogenesis, and suppression of effective T cell responses [7]. TAM infiltration has been identified as a poor prognostic factor in various cancers, including RCC [8,9]. However, the mechanism of TAM-induced RCC progression still remains unclear. Chemokines usually mediate the interaction between TAMs and cancer cells and exert various biological functions via their receptors on target cells [10]. Therefore, an approach regarding the functions of chemokines within the tumor microenvironment is thought to be reasonable for elucidating the interaction between TAMs and cancer cells.

In this study, we tested our hypothesis that TAMs in the RCC microenvironment might trigger undesirable inflammatory signals and, thereafter, could provide tumor-supporting signals to stimulate the progression of RCC. We identified CCL20, of which CCR6 is the sole receptor, as a key player in mediating Akt activation and the epithelial–mesenchymal transition (EMT) of RCC cells.

## 2. Results

### 2.1. RCC Cells Could Induce M2-Type-Like (M2L) Macrophages from M1-Type-Like (M1L) Macrophages

**Figure 1:**
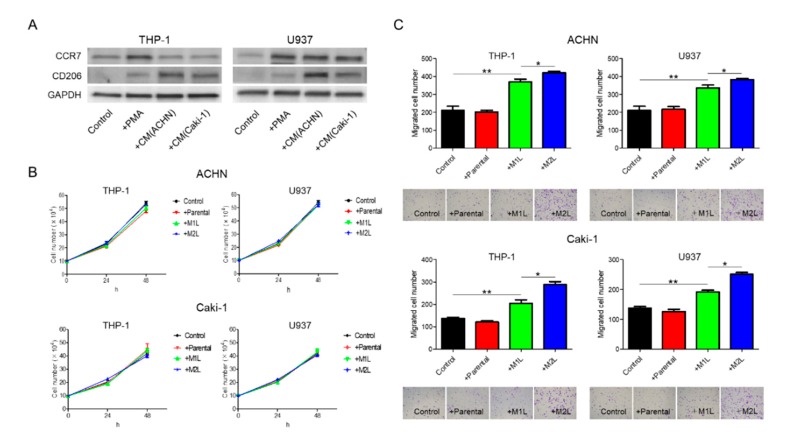
Differentiation of THP-1 and U937 cells and their effects on ACHN and Caki-1 cells. (**A**) western blotting of M1-marker, CCR7, and M2-marker, CD206 in THP-1 and U937 cells with no treatment (control), treated with 100 ng/mL of phorbol 12-myristate 13-acetate (PMA) and conditioned medium (CM) from ACHN and Caki-1 cells following PMA. THP-1 and U937 cells (2.5 × 10^5^ cells/mL) were treated with PMA for 24 h for M1-type-like (M1L) status. After PMA was removed, cells were exposed to CM of ACHN and Caki-1 cells for 4 days at 37 °C in a humidified atmosphere of 5% CO_2_ for M2-type-like (M2L) status. (**B**) Proliferation assay of ACHN and Caki-1 cells alone (control), co-cultured with THP-1 and U937 cells or M1L- and M2L macrophages. ACHN and Caki-1 cells (1 × 10^5^ cells/well) were placed into the lower compartment in 6-well plates, and parental and differentiated THP-1 or U937 cells were placed on the inserts. After co-culture for 24–48 h, ACHN and Caki-1 cells were harvested and the cells were counted. (**C**) parental and differentiated THP-1 or U937 cells were seeded in the lower compartment (no cells were seeded as control) of a 24-well plate, ACHN and Caki-1 cells were placed in the insert, and after 12 h, the cells migrated through the lower membrane were stained with crystal violet, photographed, and counted. Data are means ± SEM. All experiments were performed in triplicate. No significant difference between groups in which *t*-test performed was detected by *f*-test. * *p* < 0.05, ** *p* < 0.01.

When suspended THP-1 and U937 cells were treated with phorbol 12-myristate 13-acetate (PMA), the cells stopped proliferation, adhered to the surface of the plate, and, after 24 h, differentiated into M1L macrophage cells as we previously reported (hereinafter referred to as M1L-THP-1 and M1L-U937) [11]. While the M1L-THP-1 and M1L-U937 cells expressed the M1-type macrophage marker CCR7, they slightly express the M2-type macrophage marker CD206, indicating that both cells were not complete M1-type but had M1-type skew characteristics. After treatment with conditioned medium (CM) of ACHN or Caki-1 cells, the M1L-THP-1 and M1L-U937 cells expressed high CD206 (hereinafter referred to as M2L-THP-1 and M2L-U937), indicating that RCC cells can add some M2-type characteristics to M1L-THP-1 and M1L-U937 cells, similar to the property of TAMs (Figure 1A).

### 2.2. Macrophages Increased RCC Cell Migration

ACHN and Caki-1 cells were co-cultured with THP-1 and U937 cells, and the proliferation after 24 and 48 h and migration after 12 h were evaluated. Although there were no significant differences in the proliferation rate, irrespective of the status of the THP-1 and U937 cells, both the ACHN and Caki-1 cells showed a significant increase in migration when co-cultured with macrophage-like cells (Figure 1B,C). The migration rate of RCC cells co-cultured with M2L-THP-1 and M2L-U937 cells was significantly higher than with M1L-THP-1 and M1L-U937 cells (Figure 1C). These data indicate that M2L macrophages can induce migration but not proliferation through cell–cell interaction.

### 2.3. Macrophages Enhanced the EMT of RCC Cells

**Figure 2:**
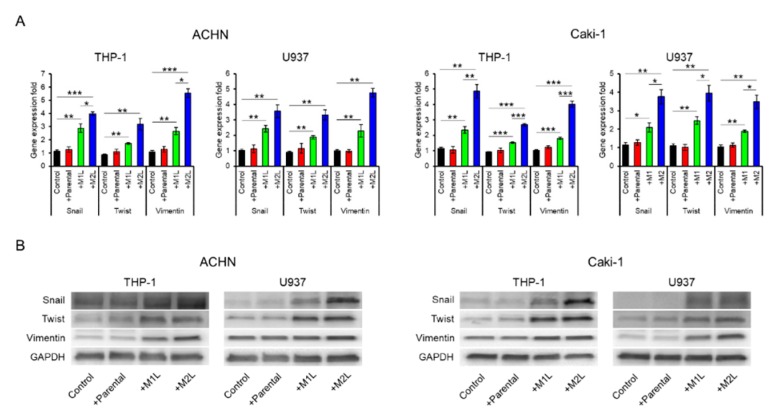
Expression of epithelial–mesenchymal transition (EMT) markers in ACHN and Caki-1 cells co-cultured with parental and differentiated THP-1 or U937 cells. (**A**) mRNA was extracted from ACHN and Caki-1 cells after co-culture (single culture as control) for 12 h, quantified, and analyzed by RT-qPCR for epithelial–mesenchymal transition markers. (**B**) Protein was extracted from ACHN and Caki-1 cells after co-culture (single culture as control) for 12 h and evaluated by western blotting. Data are means ± SEM. All experiments were performed in triplicate. No significant difference between groups in which *t*-test performed was detected by *f*-test. * *p* < 0.05, ** *p* < 0.01, and *** *p* < 0.001.

Since being co-cultured with macrophage-like cells enhanced the migration ability of ACHN and Caki-1 cells, we examined the expression of EMT-related markers. The expression levels of Snail, Twist, and Vimentin in ACHN and Caki-1 cells were significantly increased by co-culture with macrophage-like cells, especially M2L-THP-1 and M2L-U937 cells (Figure 2A). EMT-related protein levels were also increased by co-culture with macrophage-like cells (Figure 2B). These data indicate that M2L-THP-1 and M2L-U937 cells induced by the CM of RCC cells elicit cell migration through EMT change.

### 2.4. Macrophage-Like Cells Secreted CCL20

**Figure 3:**
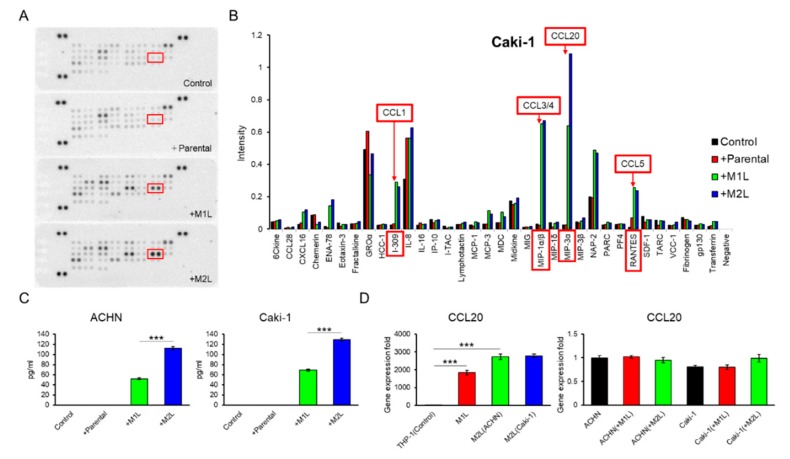
Identification and quantification of secreted chemokines that potentially induce RCC cell migration. (**A**) Membranes of a human cytokine antibody array comparing CM from Caki-1 cells alone (control), and co-cultured with parental and differentiated THP-1 cells were shown. Box indicates CCL20 spots. (**B**) The comparison of each cytokine intensity standardized by positive controls was shown. The mean values of two spots were shown. I-309, MIP-1α/β, MIP-3α, and RANTES is another name of CCL1, CCL3/4, CCL20, and CCL5, respectively. (**C**) Quantification of CCL20 concentration in CM from ACHN and Caki-1 cells alone (controls) and CM form co-culture ACHN and Caki-1 cells with parental and differentiated THP-1 cells for 12 h was determined by ELISA. (**D**) qPCR of CCL20 in parental THP-1 (control), M1L-THP-1, and M2L-THP-1 cells co-cultured with ACHN and Caki-1 cells (the left panel) and qPCR of CCL20 in ACHN (control) and Caki-1 cells alone, co-cultured with M1L-THP-1 and M2L-THP-1 cells (the right panel). Data are means ± SEM. Experiments in C and D were performed in triplicate. No significant difference between groups in which *t*-test performed was detected by *f*-test. *** *p* < 0.001.

Since THP-1-derived macrophage-like cells showed more decreased CCR7 expression in M2L-THP-1 than in M2L-U937 even both M2L-THP-1 and M2L-U937 cells stably expressed CD206 (Figure 1A), these THP-1-derived cells were focused on in the subsequent experiments. A human cytokine antibody array of CM from the co-culture of Caki-1 cells with a different status of THP-1 cells showed a high MIP-3α (CCL20) concentration in the CM of the co-culture with macrophage-like cells (Figure 3A,B). ELISA found that the amount of CCL20 secretion was proportionate to the migration effect of macrophage-like cells on ACHN and Caki-1 cells shown in Figure 1C with 0.92 and 0.99 of Pearson’s R square, respectively (Figure 3C). To examine which cells secreted CCL20 during the co-culture, qPCR was performed. The CCL20 expression levels of M1L-THP-1, M2L-THP-1 co-cultured with ACHN cells, and M2L-THP-1 co-cultured with Caki-1 cells were around 2000-, 3000-, and 3000-fold higher than that of parental THP-1 cells (Figure 3D left panel). On the other hand, the CCL20 expression levels of RCC cells were not changed when co-cultured with M1L-THP-1 and M2L-THP-1 cells (Figure 3D right panel). These qPCR data indicate that most CCL20 is potentially provided from not RCC cells but macrophage-like cells.

### 2.5. CCL20 Promoted ACHN and Caki-1 Cell Migration

**Figure 4:**
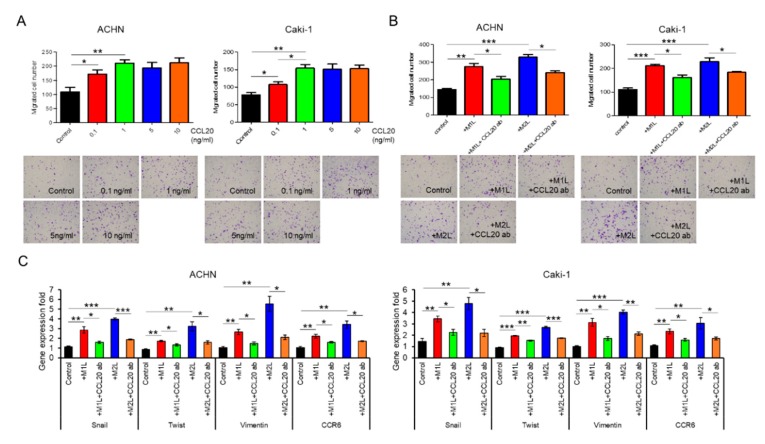
Effect of CCL20 on migration ability of ACHN and Caki-1 cells. (**A**) ACHN or Caki-1 cells were placed on inserts in 24-well plates with or without 0 (controls) −10 ng/mL human recombinant CCL20 and cell migration was assayed at after 12 h. (**B**) ACHN or Caki-1 cells were placed on inserts in 24-well plates and co-cultured with M1L- and M2L-THP-1 cells (ACHN or Caki-1 cells alone as controls) with or without anti-CCL20 neutralizing antibody (CCL20 ab) and migration was assayed after 12 h. (**C**) mRNA was extracted from ACHN and Caki-1 cells after co-cultured with M1L- and M2L-THP-1 cells (ACHN or Caki-1 cells alone as controls) with or without CCL20 ab for 12 h, quantified, and analyzed by RT-qPCR for epithelial–mesenchymal transition markers and CCR6. Data are means ± SEM. All experiments were performed in triplicate. No significant difference between groups in which *t*-test performed was detected by *f*-test. * *p* < 0.05, ** *p* < 0.01, and *** *p* < 0.001.

Although recombinant CCL20 at a concentration of 0.1–1.0 ng/mL increased ACHN and Caki-1 cell migration in a dose-dependent manner, no additional effect of a high concentration of CCL20 over 1.0 ng/mL was found (Figure 4A). Since secretions of I-309 (CCL1), MIP-1α/β (CCL3/4), and RANTES (CCL5) were also specifically increased in the CM of the co-culture with macrophage-like cells in the human cytokine antibody array, these four chemokines were thought of as potential factors for the activation of RCC cells. The migration of ACHN and Caki-1 cells was evaluated using similar amounts of these four recombinant chemokines but was not induced by them (Supplementary Figure S1). To confirm that CCL20 can activate RCC cells, anti-CCL20 neutralizing antibody was added to ACHN and Caki-1 cells co-cultured with macrophage-like cells, and a significant inhibition of cell migration was observed in both the ACHN and Caki-1 cells (Figure 4B). The upregulated expression levels of Snail, Twist, and Vimentin in ACHN and Caki-1 cells co-cultured with macrophage-like cells were significantly decreased by the anti-CCL20 neutralizing antibody (Figure 4C). Interestingly, the expression of CCR6, which is the sole receptor of CCL2, was decreased by the anti-CCL20 neutralizing antibody as well (Figure 4C). These data indicate that the CCL20-CCR6 axis is an upstream of EMT markers and that depletion of the ligand induces CCR6 downregulation.

### 2.6. CCL20-CCR6 Axis Activated Akt

**Figure 5:**
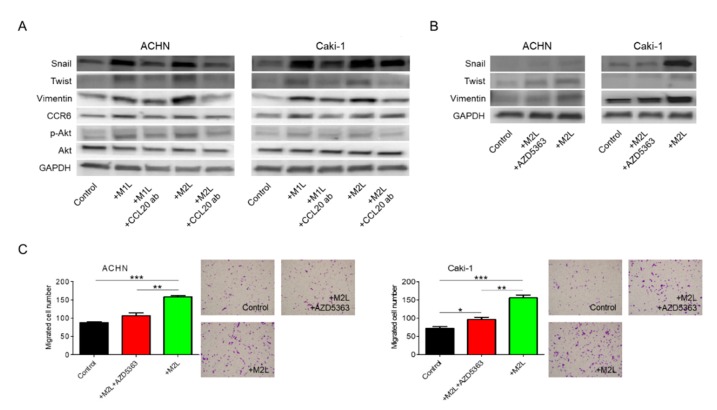
Involvement of Akt phosphorylation in EMT change induced by CCL20-CCR6 axis. (**A**) Protein was extracted from ACHN and Caki-1 cells after co-cultured with M1L- and M2L-THP-1 cells (ACHN or Caki-1 cells alone as controls) with or without anti-CCL20 neutralizing antibody, and EMT, CCR6, and phosphorylation of Akt were evaluated by western blotting. (**B**) Protein was extracted from ACHN and Caki-1 cells after co-culture with M2L-THP-1 cells (ACHN or Caki-1 cells alone as controls) with or without the Akt inhibitor, AZD5363, for 12 h and EMT were evaluated by western blotting. (**C**) ACHN or Caki-1 cells were placed on inserts in 24-well plates with M2L-THP-1 cells (ACHN or Caki-1 cells alone as controls) with or without AZD5363 and migration was assayed after 12 h. Data are means ± SEM. All experiments were performed in triplicate. No significant difference between groups in which *t*-test performed was detected by *f*-test. * *p* < 0.05, ** *p* < 0.01, and *** *p* < 0.001.

We have previously shown that chemokines increase cell migration and invasiveness via Akt activation in prostate cancer [10,11,12]. Thus, Akt, which is a well-known cancer-cell-activating pathway, was focused on as a potential downstream effector of the CCL20-CCR6 axis in the migration of RCC cells. Administration of anti-CCL20 neutralizing antibody reduced macrophage-like cell-induced upregulation of EMT markers and phosphorylation of Akt in RCC cells (Figure 5A). Interestingly, CCR6 was also controlled by its ligand, CCL20. When Akt inhibitor AZD5363 was added to M2L-THP-1 and M2L-U937 cells, the increase of EMT markers was suppressed (Figure 5B). To determine whether activation of Akt is responsible for the increased migratory capacity of RCC cells, AZD5363 was used in the migration assay of RCC cells. AZD5363 could significantly suppress RCC cell migration co-cultured with macrophage-like cells (Figure 5C). These results clearly indicate that Akt is a downstream target of the CCL20-CCR6 axis and an important pathway in promoting RCC cell migration.

### 2.7. CCR6 Expression is a Poor Prognostic Factor in RCC

**Figure 6:**
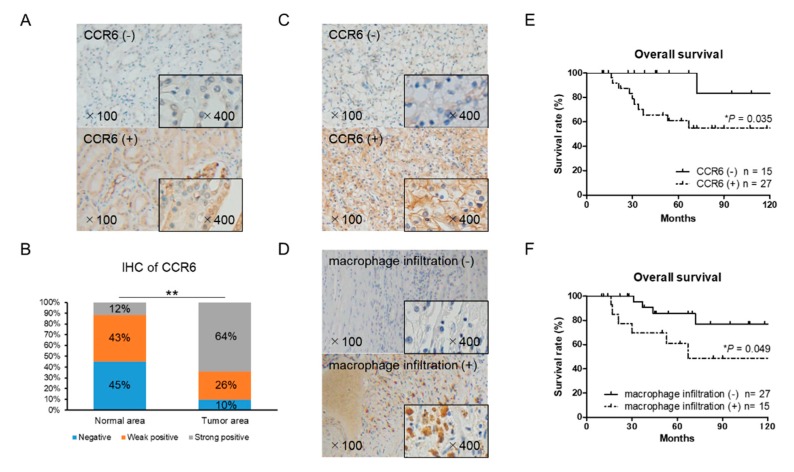
Immunohistochemical staining of CCR6 and CD68 using 42 RCC tissue samples. (**A**) Normal (non-cancer) areas in RCC tissues were stained with anti-CCR6 antibody. Negative and positive staining samples of normal area were shown (the upper and lower panel, respectively). (**B**) The distribution of CCR6 staining intensity in normal and tumor area showed stronger expression of CCR6 in tumor area than in normal area. The χ^2^-test for trend was performed. (**C**) Tumor area of RCC tissues was stained with anti-CCR6 antibody. The upper and lower panel shows a negative and positive staining sample, respectively. (**D**) Tumor area of RCC tissues was stained with anti-CD68 antibody to detect infiltrating macrophages into RCC tumor microenvironment. The upper and lower panel shows a negative and positive staining sample, respectively. (**E**) Overall survival of CCR6 positive (*n* = 27) and negative (*n* = 15) patients are shown. (**F**) Overall survival of CD68 positive (*n* = 15) and negative (*n* = 27) patients are shown. Kaplan–Meier curves were used to estimate survival distributions and the log-rank test was used to analyze survival differences. * *p* < 0.05 and ** *p* < 0.01.

The expression of CCR6 and macrophage marker CD68 in RCC tissues was examined by immunohistochemistry. There was a significant difference in CCR6 staining between the cancer and normal areas in the RCC tissues (Figure 6A,B). CCR6 positive cells were observed in 27 of the 42 RCC tissues (Figure 6C). Infiltration of CD68 positive macrophages into tumor tissue was observed in 15 of 42 RCC tissues (Figure 6D). The expression level of CCR6, but not CD68, was significantly higher in the ≥T2, N+, and N+ samples than in the T1 localized small tumor samples (Table 1). Importantly, the overall survival of CCR6 positive patients was significantly poorer than that of negative patients (*p* = 0.035) (Figure 6E). Although, there was no significant association between CCR6 positive and CD68 positive patients, the overall survival of CD68 positive patients was significantly poorer than that of CD68 negative patients (*p* = 0.049) (Figure 6F). These clinical data on CCR6 and macrophage infiltration that indicate poor prognoses in RCC patients are consistent with in vitro results and strongly support the important role of infiltrating macrophages and the CCL20-CCR6 axis in RCC progression.

## 3. Discussion

Immune cell infiltration is a common feature of most types of cancer, and macrophages play an important role in the interaction between the immune system and cancer cells [13]. Macrophages in the tumor microenvironment are called TAMs and usually show M2-type characteristics [14]. TAMs are known to promote tumor tissue angiogenesis, immunosuppression, proliferation, invasiveness, and metastasis [15,16]. In our in vitro study, macrophage-like cells similar to TAMs enhanced RCC cell migration. Among the various immune factors involving mechanisms of TAM-induced cancer cell migration, chemokines have been reported as critical factors for the promotion of migration in cancers [11,17,18]. Therefore, we focused on chemokines as a contributing factor to tumor promotion in RCC cells. A human cytokine antibody array of CM from a co-culture of Caki-1 cells with a different status of THP-1 cells showed a high CCL20 concentration in the CM of the co-culture with macrophage-like cells. More importantly, CCL20 alone showed different secretion levels between M1L- and M2L-macrophages among the chemokines increased in the CM of the co-culture with macrophage-like cells. This difference of CCL20 secretion was proportionate to the migration effect of macrophage-like cells on RCC cells. Although other chemokines might potentially have some effects on RCC cells, whether positive or negative, CCL20 could be regarded as a key chemokine that directly modulates the behaviors of RCC cells. Finally, we found that the CCL20-CCR6 axis greatly contributes to the promotion of migration in RCC cells with suppression of the axis.

Several studies have revealed the role of CCL20 and its specific receptor, CCR6 [19,20]. As our data indicate, TAMs likely secrete CCL20, and CCR6-expressed RCC cells consume CCL20 in the tumor microenvironment of RCC, resulting in an increased migration ability of the RCC cells. In RCC, CCR6 was studied in relation to T lymphocyte infiltration into tumor tissue, but no relationship was found between the expression level of CCR6 and the number of infiltrated T lymphocytes [21]. Indeed, the role of CCR6 in RCC cells has yet to be evaluated. The present study clearly showed for the first time that the CCL20-CCR6 axis in macrophage-RCC cell interaction can induce Akt activation and subsequent EMT in RCC cells. Consistently, Akt activation has been reported as a downstream of the CCL20-CCR6 axis [22], and EMT change induced by Akt activation though the CCL20-CCR6 axis has been reported in gastric cancer and cervical cancer cells [23,24].

Furthermore, the RCC tissue specimens showed that the high expression level of CCR6 is not only associated with the advanced status of RCC but also significantly shortens overall survival in RCC patients. There was no association in expression of CCR6 and CD68 positive tissues. CD68 positive cells in RCC tissues are not necessarily TAMs secreting CCL20 and do not necessarily induce CCR6 on RCC cells. These may be reasons of inconsistency of CCR6 positive and CD68 positive tissues. The CCL20-CCR6 axis may be a novel biomarker as well as a novel treatment target for RCC. Serum chemokine concentration and the expression level of tissue chemokine receptor have been reported as predictive biomarkers of survival in cancer patients [25,26]. As there are no definite serum biomarkers for RCC to date, specific and effective biomarkers need to be developed.

Some other points must be discussed carefully in this study. The chemokine array showed high intensity in some chemokines. Although we did not focus on these chemokines in detail because no significant difference in the co-cultured CM between M1L- and M2L-macrophages was observed on array, they may exert some effects on RCC cells in cooperation with other functional molecules. Next, no definite difference between M1L- and M2L-macrophages was confirmed. To begin with, M1- and M2-types cannot be separated clearly, and a consecutive change of character might exist between them. Differentiation of macrophages in this study may be insufficient since several techniques/methods were reported to differentiate M1- and M2-type macrophages completely [27]. The critical status contributing to CCL20 secretion needs to be clarified to further develop this study. Moreover, the clinical sample size was too small to assess the role of CCR6 in RCC. Additional clinical evidence is needed to confirm the availability of the CCL20-CCR6 axis as an RCC biomarker.

## 4. Materials and Methods

### 4.1. Reagents and Antibodies

Recombinant human CCL20 (360-MP) was purchased from R&D Systems (Minneapolis, MN, USA). The following antibodies were used in western blot analyses, neutralizing assays, and immunohistochemistry: rabbit anti-CCL20 (ab9829), rabbit anti-CCR6 (ab227036), rabbit anti-CCR7 (ab103404), rabbit anti-CD206 (ab64693), mouse anti-CD68 (ab31630), mouse anti-Snail (ab117866), mouse anti-Twist (ab175430), and rabbit anti-Vimentin (ab92547), from Abcam (Cambridge, MA, USA); rabbit anti-Akt (9272S), rabbit anti-phospho-Akt (Ser473; 9271S), and HRP-conjugated anti-rabbit IgG (7074) antibodies from Cell Signaling Technology (Danvers, MA, USA); mouse anti-GAPDH (NB300-221) antibody from Novus Biologicals (Littleton, CO, USA); and HRP-conjugated anti-mouse IgG (1706516) antibody from Bio-Rad Laboratories (Hercules, CA, USA). The Akt inhibitor AZD5363 (S8019) was purchased from Selleck Chemicals (Houston, TX, USA).

### 4.2. Cell Culture

ACHN and Caki-1 human RCC cell line cells and THP-1 and U937 human monocytic leukemia cell line cells were purchased from the American Type Culture Collection (Manassas, VA, USA). All cells were maintained in a growth medium containing RPMI-1640 (Thermo Fisher Scientific, Waltham, MA, USA) supplemented with 10% FBS and 1% penicillin/streptomycin (Invitrogen, Carlsbad, CA, USA) and kept at 37 °C in a humidified atmosphere with 5% CO_2_.

### 4.3. Conditioned Medium (CM)

A total of 1 × 10^5^ ACHN and Caki-1 cells were seeded into six-well culture plates and allowed to adhere overnight. The next day, the medium was aspirated and replaced with 2 mL of medium containing 10% FBS. The supernatant was removed 24 h later, centrifuged at 400 g for 10 min, and preserved as CM.

### 4.4. Preparation of Macrophage-Like Cells

The THP-1 and U937 cells were used as models for monocyte-macrophage differentiation. When collecting these cells, medium containing floating cells was centrifuged at 800 rpm for 10 min, and the cell number was counted using a hemocytometer. M2L-macrophages were generated from monocytic parental THP-1 and U937 cells as described previously [11]. Briefly, the THP-1 and U937 cells (2.5 × 10^5^ cells/mL) were treated with 100ng/mL of PMA for 24 h (M1L status). After the cells stopped growing and adhered to the surface of the plate, they were washed three times with PBS to remove the PMA and then exposed to the CM of the ACHN and Caki-1 cells for four days at 37 °C in a humidified atmosphere of 5% CO_2_ (M2L status).

### 4.5. Co-Culture Assay

Co-culture experiments were performed using cell culture inserts (Falcon, Corning, NY, USA) in 6-well or 24-well plates. For extraction of protein and RNA, 1 × 10^5^ ACHN, Caki-1, parental THP-1, U937 cells, and M1- or M2-polarized THP-1, U937 cells were seeded in the lower compartment of six-well plates. The plates were incubated for six hours for cell attachment. Thereafter, the same number of affecting co-culture cells were seeded in cell culture inserts with 1.0 μm pore size (cells cannot move through pores), and cell culture inserts were placed in the lower compartment and incubated for the appropriate amount of time and if needed, reagents were added to the lower compartment. The cells placed in the lower compartment were collected for extraction of protein and RNA. Detailed extraction methods are described further on.

### 4.6. Proliferation Assays

In the cell proliferation assays, 1 × 10^5^ ACHN or Caki-1 cells were seeded in the lower compartment of six-well plates first. The plates were incubated for six hours for cell attachment. Thereafter, the same number of parental THP-1 and U937 cells or M1L-, M2L-THP-1, M1L-, and M2L-U937 cells were seeded in cell culture inserts with 1.0 μm pore size (cells cannot move through pores), and cell culture inserts were placed in the lower compartment and incubated for 24 and 48 h. The ACHN or Caki-1 cells were trypsinized, harvested, and counted using a hemocytometer.

### 4.7. Cell Migration Assays

Cell migration assays were also performed using cell culture inserts with 8.0 μm pore size (cells can migrate through pores) placed in 24-well plates. The ACHN and Caki-1 cells were grown to 80% confluence in growth medium, and 3 × 10^3^ cells were seeded on the cell culture inserts. Subsequently, the cells were cultured in RPMI-1640 containing 0.1% FBS for six hours at 37 °C in a humidified incubator containing 5% CO_2_ for starvation. When starting the cell migration assay, 7 × 10^3^ THP-1 and U937 cells or M1L-, M2L-THP-1, M1L-, and M2L-U937 cells were seeded in the lower compartment. Recombinant CCL20, anti-CCL20 neutralizing antibody, and Akt inhibitor were appropriately added in the lower compartment. The lower compartment was administered the RPMI-1640 medium containing 10% FBS. The ACHN and Caki-1 cells were incubated for 12 h at 37 °C in a humidified incubator containing 5% CO_2_. Then, the cells on the filter of cell culture inserts were fixed with 4% paraformaldehyde in PBS for 10 min. The cells on top of the filter were carefully removed with a cotton swab, and the cells on the back side of the filter were stained with 0.1% crystal violet for 15 min. The stained filter was microscopically photographed, and the cell number was counted.

### 4.8. RNA Extraction and RT-qPCR Analysis

Total RNA was purified with the RNA Isolation Kit (Zymo Research, Irvine, CA, USA), following the manufacturer’s instructions. The RNA concentration was measured using a NanoDrop spectrophotometer (Thermo Fisher Scientific). First-strand cDNA was prepared from an RNA template (500 ng) using the iScript cDNA Synthesis Kit (Bio-Rad). Reverse transcription was performed at 25 °C for 5 min, at 46 °C for 20 min, and then at 95 °C for 1 min. qPCR was performed using the CFX Connect ^TM^ Real-Time PCR Detection System (Bio-Rad). RT-qPCR was performed using the SsoAdvanced Universal SYBR Green Supermix (Bio-Rad) to detect gene expression. The first step was 95 °C for 3 min; the second step was 95 °C for 15 s and 60 °C for 30 s, repeated 40 times. Each gene was normalized with GAPDH and analyzed. The primer sequences are shown in Table 2.

### 4.9. Western Blot Analysis

Cell lysates were prepared using M-PER mammalian protein (Thermo Fisher Scientific) containing 1% protease inhibitor cocktail and phosphatase inhibitor cocktail (Sigma-Aldrich, St. Louis, MO, USA). Soluble lysates (10–20 μg) were mixed with a lithium dodecyl sulfate sample buffer and sample reducing agent, both obtained from Thermo Fisher Scientific, and separated by SDS-PAGE. The separated proteins were transferred to nitrocellulose membranes. The membranes were blocked with 1% gelatin and 0.05% Tween in Tris-buffered saline for one hour at room temperature. The membranes were then incubated overnight at 4 °C with primary antibody according to the manufacturer’s instructions. After washing, the membranes were incubated with HRP-conjugated anti-rabbit or anti-mouse secondary antibody for one hour at room temperature. Protein bands were detected using the Super Signal West Femto maximum sensitivity substrate (Thermo Fisher Scientific).

### 4.10. Chemokine Array

Chemokines were assayed using CM collected from Caki-1 co-cultured with parental, M1L-, and M2L-THP-1 cells using a Human Chemokine Array Kit (ARY017, R&D Systems), following the manufacturer’s instructions.

### 4.11. Enzyme-Linked Immunosorbent Assay (ELISA)

Human CCL20 secretion was measured in the CM from ACHN or Caki-1 cells and the co-culture CM of co-cultured ACHN or Caki-1 cells and parental, M1L-, and M2L-THP-1 cells using a Human MIP-3α Quantikine ELISA Kit (DM3A00, R&D Systems), following the manufacturer’s instructions. MIP-3α is another name for CCL20. The absorbance was measured at 450 nm and corrected at 540 nm on a microplate reader.

### 4.12. Immunohistochemistry

A total of 42 RCC tissue samples, including 32 non-metastatic and 10 metastatic, were obtained from patients who underwent a nephrectomy or partial nephrectomy at Kanazawa University Hospital (Kanazawa, Japan). The study plan was approved by the Medical Ethics Committee of Kanazawa University. The paraffin block specimens were cut at a thickness of 3 μm and deparaffinized. For antigen activation, slides sunk in 20 mM Tris/20 mM HCl (pH 9.0) were heated for 20 min at 95 °C (sub-boiling temperature) and cooled on a bench top for 30 min. The slides were incubated for 10 min at room temperature in 0.3% H_2_O_2_ methanol to suppress endogenous peroxidase activity. The slides were blocked with 3% bovine serum albumin in PBS for 30 min at room temperature. Then, the primary antibody diluted in PBS containing 1% bovine serum albumin was applied, and the slides were incubated overnight at 4 °C. The slides were thoroughly washed with PBS; HRP-conjugated secondary antibody was added, and they were incubated at room temperature for one hour. Finally, the slides were developed with ImmPACT DAB EqV Peroxidase Substrate (Vector Laboratories, Burlingame, CA, USA) for 5 m at room temperature, and then counter staining was performed with hematoxylin. The CCR6 staining intensity was recorded as positive (10% or more than 10%), weak positive (less than 10%), or negative (none), and the CD68 staining was recorded as positive or negative.

### 4.13. Statistical Analyses

Statistical analyses were performed using the commercially available software GraphPad Prism (GraphPad Software, San Diego, CA, USA). The Student’s *t*-test was used to assess between-group differences with *f*-test for comparing between-group variances. Pearson test was used to evaluate the correlation between two data sets. The Fisher’s exact-test was used to evaluate the relationship between two variables. Kaplan–Meier curves were used to estimate survival distributions. The log-rank test was used to analyze survival differences. Significance was defined as * *p* < 0.05, ** *p* < 0.01, and *** *p* < 0.001.

## 5. Conclusions

To our knowledge, this is the first study to show that the CCL20-CCR6 axis contributes to RCC cell migration and may be a novel therapeutic target and potential biomarker for RCC.

## Figures and Tables

**Table 1:** CCR6 expression and macrophage infiltration in tissue samples.

			CCR6			CD68		
Parameters		*n*	Pos	Neg *	*p*	Pos	Neg	*p*
Gender	Male	31	20	11	n.s.	12	19	n.s.
	Female	11	7	4		3	8	
Age	≤60	16	9	7	n.s.	6	10	n.s.
	>60	26	18	8		9	17	
Histology	Pure CC	37	24	13	n.s.	13	24	n.s.
	CC with SC	5	3	2		2	3	
TNM	T1N0M0	16	6	10	0.008	7	9	n.s.
	Other	26	21	5		8	18	

Pos, positive; Neg, negative; CC, clear cell type; SC, sarcomatoid change; T, tumor; N, node; M, metastasis; * includes weak positive.

**Table 2:** Primer sequences using for qPCR.

GAPDH	forward	5′-TCT CTG CTC CTC CTG TTC GA-3′
	reverse	5′-GCG CCC AAT ACG ACC AAA TC-3′
Snail	forward	5′-ATG GCC ATT TCT GTG GAG GG-3′
	reverse	5′-CAA AAA CCC ACG CAG ACA GG-3′
Twist	forward	5′-ATT CAG ACC CTC AAG CTG GC-3′
	reverse	5′-AGC TTG CCA TCT TGG AGT CC-3′
Vimentin	forward	5′-AAC TTA GGG GCG CTC TTG TC-3′
	reverse	5′-ATT CAA GTC TCA GCG GGC TC-3′
CCR6	forward	5′-AAT CGC TTG AAC CCA GAA GG-3′
	reverse	5′-GAG TCT CGC TTT GTC ACC CA-3′
CCL20	forward	5′-GGA ATG GAA TTG GAC ATA GCC-3′
	reverse	5′-CCT CCA TGA TGT GCA AGT GA-3′

## Supplementary Materials

The following are available online at https://www.mdpi.com/2072-6694/12/1/89/s1, Figure S1: The migration of ACHN (A) and Caki-1 (B) treated with four potential chemokines. Migration was not induced by any chemokines.
