# Supplementary material for: Tumor-Associated Macrophages Induce Migration of Renal Cell Carcinoma Cells via Activation of the CCL20-CCR6 Axis
Source: Cancers (Basel). 2019 Dec 30;12(1):89. doi: 10.3390/cancers12010089 (PMC7017081; doi:10.3390/cancers12010089)
Supplement: Supplementary file 1 [file cancers-12-00089-s001.pdf]

Supplementary Materials: Tumor-associated macrophages induce migration of renal cell carcinoma cells via activation of the CCL20-CCR6 axis

Suguru Kadomoto, Kouji Izumi, Kaoru Hiratsuka, Taito Nakano, Renato Naito, Tomoyuki Makino, Hiroaki Iwamoto, Hiroshi Yaegashi, Kazuyoshi Shigehara, Yoshifumi Kadono, Hiroki Nakata, Yohei Saito, Kyoko Nakagawa-Goto, and Atsushi Mizokami

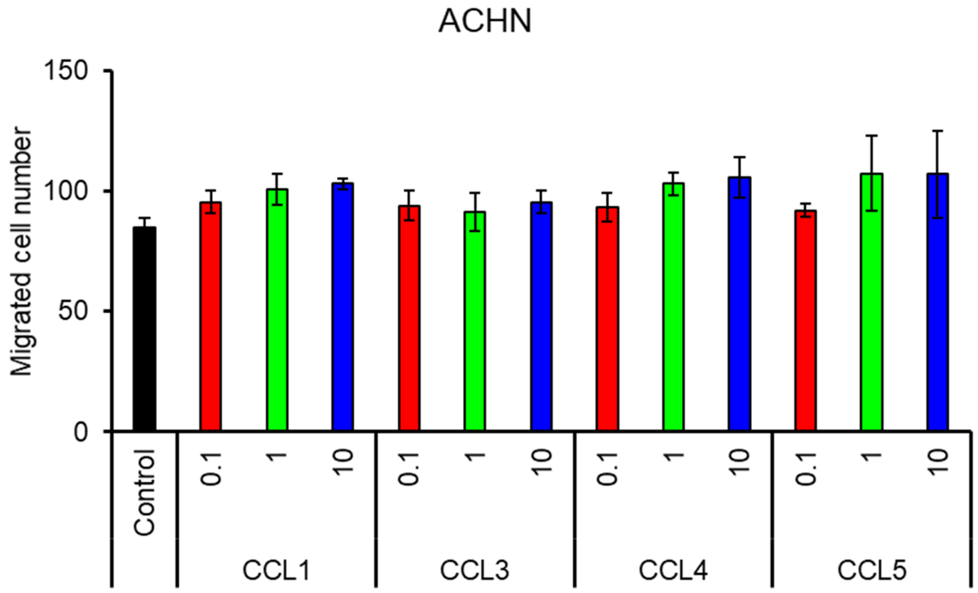

(**A**)

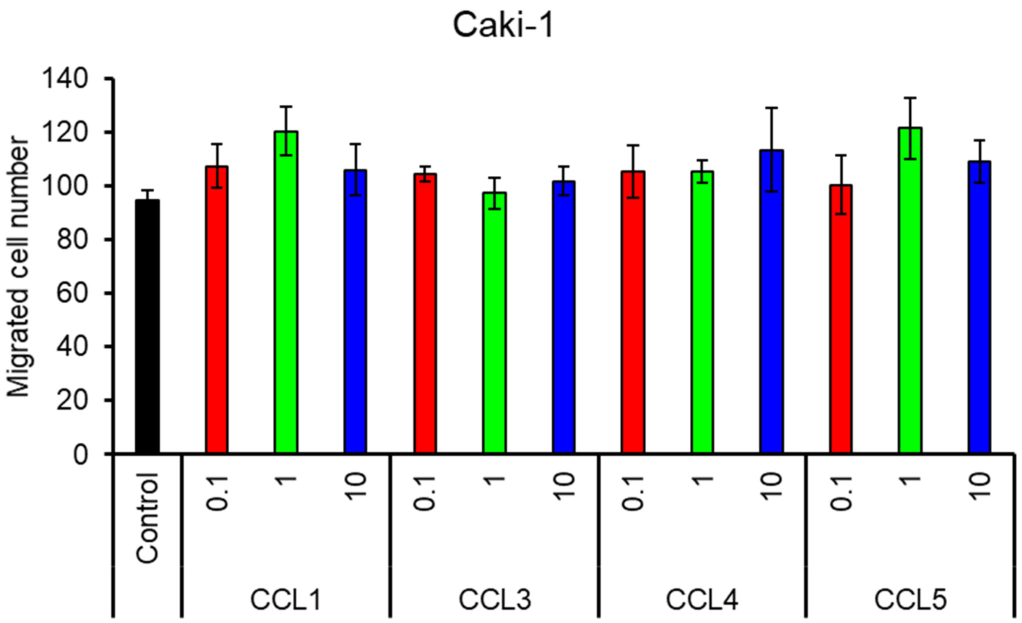

(**B**)

Figure S1. The migration of ACHN (A) and Caki-1 (B) treated with four potential chemokines. migration was not induced by any chemokines. Recombinant human CCL1 (272-I), CCL3 (270-LD), CCL4 (8228-MB), and CCL5 (278-RN) were purchased from R&D Systems (Minneapolis, MN, USA).

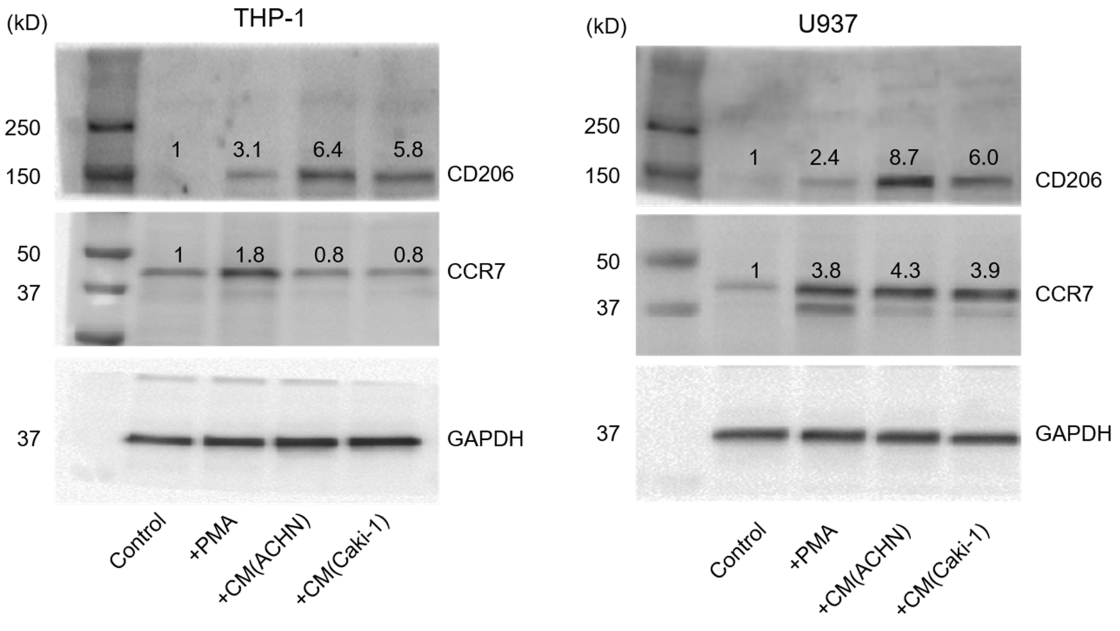

Figure S2. Full blot of Figure 1A.

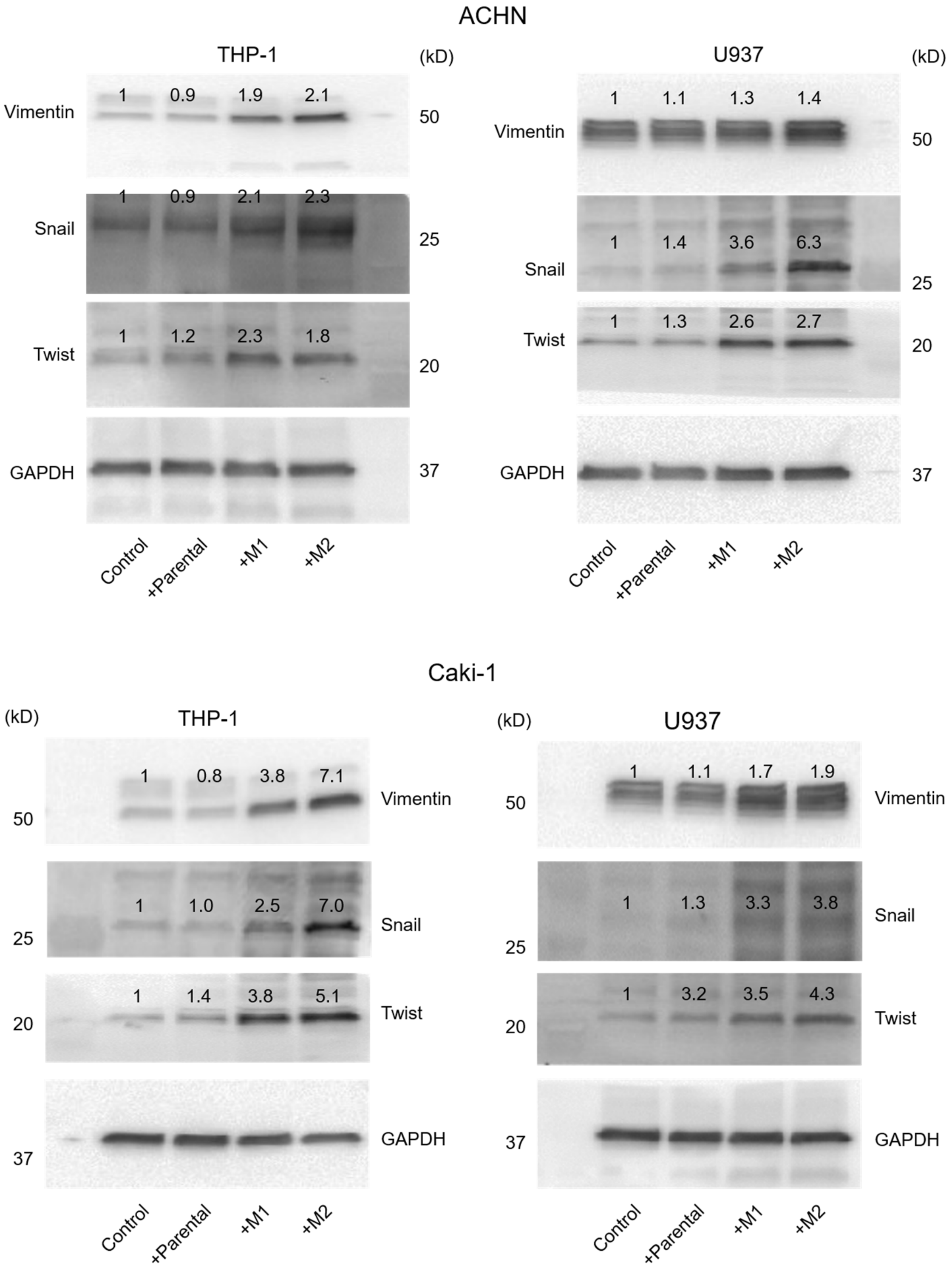

Figure S3. Full blot of Figure 2B.

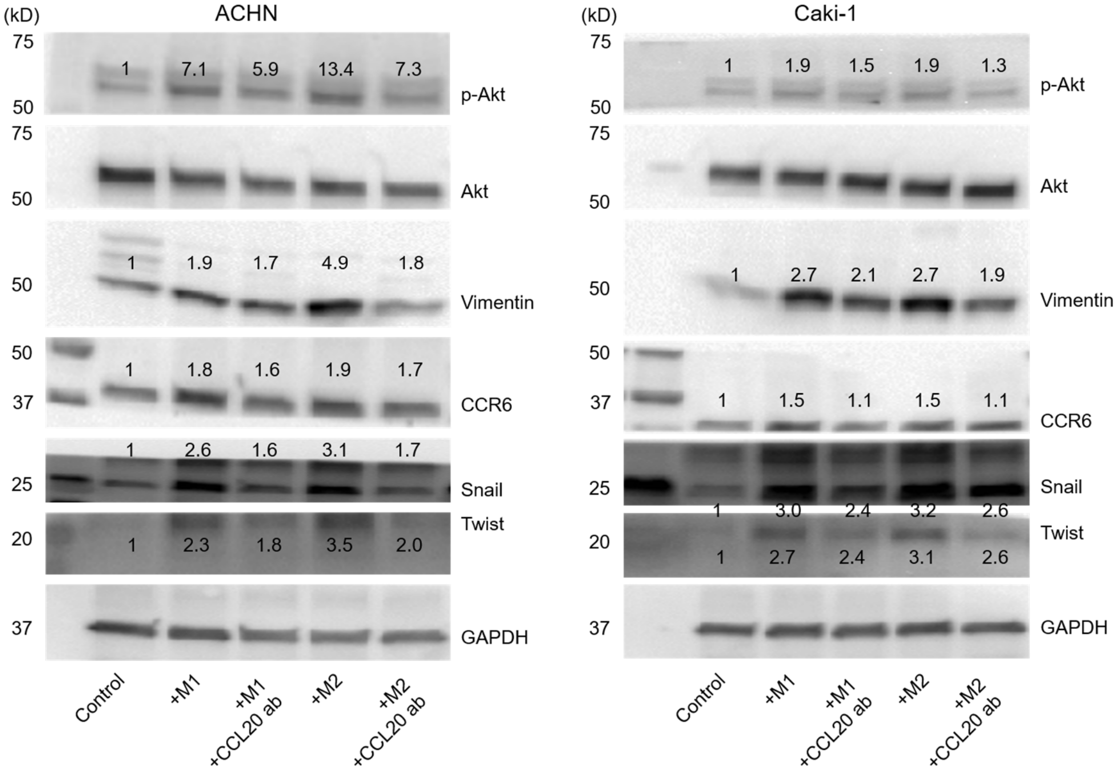

Figure S4. Full blot of Figure 5A.

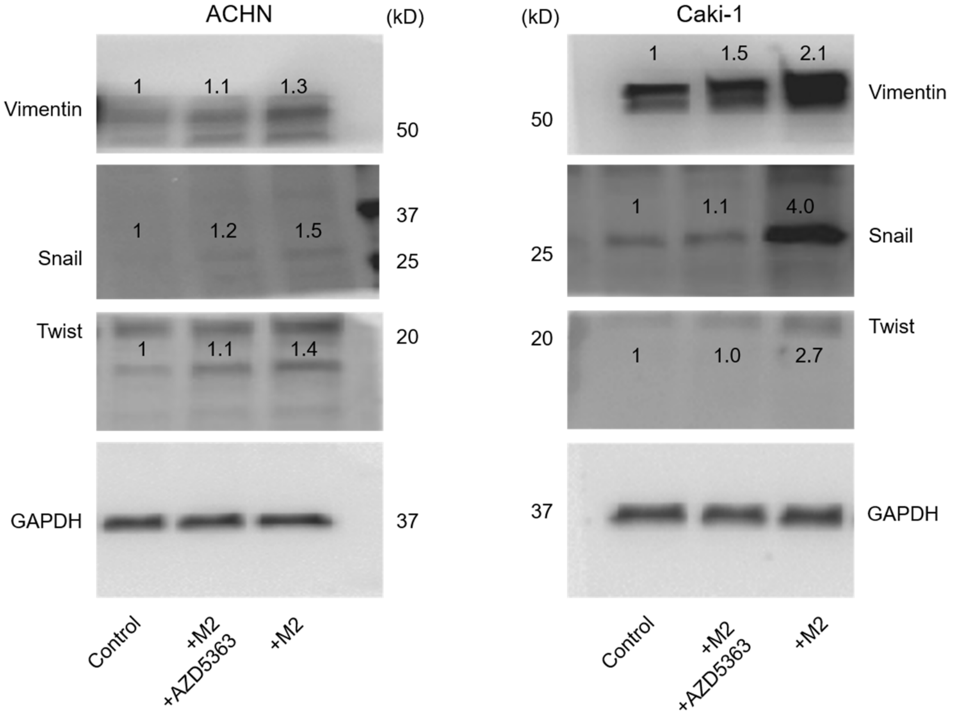

Figure S5. Full blot of Figure 5B.
